# Proximal femoral nail anti-rotation versus cementless bipolar hemiarthroplasty for unstable femoral intertrochanteric fracture in the elderly: a retrospective study

**DOI:** 10.1186/s12891-019-2793-8

**Published:** 2019-10-29

**Authors:** Shenghu Zhou, Jun Liu, Ping Zhen, Weiwei Shen, Yanfeng Chang, Haoqiang Zhang, Qingsheng Zhu, Xusheng Li

**Affiliations:** 1 Department of Joint Surgery, Institute of Orthopedics, Xijing Hospital of Fourth Military Medical University, Xi’an, 710032 China; 2 Department of Joint Surgery, Institute of Orthopedics, General Hospital of Lanzhou Military Command, Lanzhou, 730050 China

**Keywords:** Femoral intertrochanteric fracture, Elderly, Proximal femoral nail anti-rotation, Hemiarthroplasty

## Abstract

**Background:**

The treatment for unstable intertrochanteric fractures in the elderly has always been a controversial issue. The aim in this study was to compare the curative effects of proximal femoral nail anti-rotation (PFNA) and cementless bipolar hemiarthroplasty (CPH) on femoral intertrochanteric fracture in the elderly.

**Methods:**

From March 2008 to December 2012, 108 elderly patients with femoral intertrochanteric fractures were treated by PFNA or CPH. There were 63 males and 45 females, aged 75.3–99.1 years [(83.7 ± 5.6) years]. The patients’ bone mineral density was routinely measured, and the fractures were classified according to Evans-Jensen. The patients were divided into CPH group and PFNA group. The differences in operation time, intraoperative bleeding, immobilization duration, hospitalization time, Harris scores and postoperative complications including deep venous thrombosis, lung and urinary infection were analyzed.

**Results:**

All patients were followed for 12.5–36.2 months [(28.0 ± 6.3) months)]. The operation time was (53.7 ± 15.2) min and (77.5 ± 16.8) min in PFNA group and CPH group, respectively (*P* < 0.05); intraoperative bleeding was (132.5 ± 33.2) mL and (286.3 ± 43.2) mL, respectively (*P* < 0.05); immobilization duration was (28.2 ± 3.7) days and (3.1 ± 1.2) days, respectively (*P* < 0.05); hospitalization time was (7.6 ± 1.8) days and (6.9 ± 2.2) days, respectively (*P* > 0.05); and the Harris scores after 1 year were (87.7 ± 7.9) points and (88.3 ± 9.2) points, respectively (*P* > 0.05). There was no significant difference in postoperative complications between the two groups (*P* > 0.05).

**Conclusion:**

Both PFNA and CPH are safe and effective treatments for femoral intertrochanteric fracture in elderly patients. Nonetheless, CPH allows faster mobilization and recovery.

**Trial registration:**

Registration Number: ChiCTR1900022846.

Reg Date:2019-04-26 00:27:33

Retrospective registration

## Background

In the current aging society, the incidence of hip fracture in elderly is increasing every year [1]. Intertrochanteric fracture accounts for 50% of hip fractures, and the mortality within one year after the fracture is as high as 15 to 20% [2]. Because hip fractures in elderly patients are often accompanied by underlying diseases such as severe osteoporosis, hypertension, diabetes, and chronic lung disease, patients often have a poor general condition and low surgical tolerance. Thus, they are prone to bedrest-related complications after surgery. The treatment for unstable intertrochanteric fractures in the elderly has always been controversial. Most authors advocate the use of proximal femoral nail anti-rotation (PFNA) with a type of intramedullary nailing (IMN), for the treatment of unstable intertrochanteric femoral fractures [3]. However, in latest years, some authors have suggested the use of cementless bipolar hemiarthroplasty (CPH) to treat unstable intertrochanteric fractures, and satisfactory results have been achieved [4]. In this study, we retrospectively analyzed the clinical efficacy and safety of CPH and PFNA for treating unstable intertrochanteric fractures inpatients older than 75 years of age from March 2008 to December 2012 in our hospital.

## Methods

### General information

Inclusion criteria: (1) patients with type III-V intertrochanteric fracture according to the Evans-Jensen classification [5, 2) patients over 75 years; (3) patients with a fracture that occurred after a low energy trauma; and (4) patients with severe osteoporosis (T < -2.5 SD). Exclusion criteria: (1) the presence of mental disorders; (2) multiple organ dysfunctions. There involved 108 elderly patients with unstable femoral intertrochanteric fractures admitted to Department of Joint Surgery, Institute of Orthopedics, General Hospital of Lanzhou Military Command from March 2008 to December 2012. There were 63 males and 45 females, with a mean age of 75.3–99.1 years [(83.7 ± 5.6) years].

All 108 patients underwent routine preoperative bone density testing by dual-energy X-ray film in order to explore the extent of osteoporosis. Conventional anteroposterior and lateral pelvis X-ray examinations and Evans-Jensen classification were conducted [5]. Before the patients chose their treatment option according to their wishes, a medical professional gave them a full explanation of both options. Of all the patients, 47 patients were treated with CPH [CPH group, including 17 type III fractures, 19 type IV fractures, and 11 type V fractures; including 30 grade III, and 17 grade IV according to American Society of Anesthesiologist (ASA)] and 61 patients treated with PFNA (PFNA group, including 27 type III fractures, 22 type IV fractures, and 12 type V fractures; including 42 ASA grade III and 19 ASA grade IV) based on treatment methods. There was no significant difference in the general data, such as fracture type, gender and age, between the two groups (*P* > 0.05). All the patients were combined with different degrees of diseases such as hypertension, diabetes, hypoxemia, and chronic lung disease; therefore, ASA grades of the patients were recorded. All the patients were actively treated preoperatively for their underlying diseases by relevant medical consultation, and the operation was not delayed until the comprehensive assessment showed that they could tolerate surgery. All patients or their families signed the informed consent before surgery. The study was approved by the Ethics Committee of General Hospital of Lanzhou Military Command.

### Surgical procedures

#### CPH group

All 47 patients underwent nerve block or spinal anesthesia in the supine position with the contralateral healthy hip fixed to maintain positioning. During the operation, a lateral hip approach was implemented: layer-by-layer incisions were made to expose the fracture site, the joint capsule was cut, femoral neck osteotomy was performed, and the femoral trochanter fractures were reduced and fixed with cerclage wire. Medullary cavity burs were used to expand the medullary cavity. A suitable biological long-stem femoral prosthesis was selected according to the preoperative X-ray measurement and the actual intraoperative status of the medullary cavity. The anteversion angle of the femoral stem was maintained at 15°-20°, the femoral head model was inserted, and the hip joint was reduced. The stability of the reduction was tested intraoperatively to measure the vertical offset, horizontal offset and limb length of the hip joint after ensuring the absence of dislocation. These values were compared for the preoperative assessment. After satisfactory results were obtained, the corresponding femoral prosthesis and the femoral bipolar head were implanted before being reduced. The external hip rotators and rear composite hip were sutured for irrigation and suction drainage. The biotype artificial joint was provided by the Zimmer Company (United States) and the Link Company (Germany).

#### PFNA group

The 61 patients in the PFNA fixation group underwent nerve block or spinal anesthesia and were placed in a supine position. They were placed in the traction bed, the ipsilateral hip was internally rotated to 15°, and the intertrochanteric fracture was reset under C-arm fluoroscopy guidance [6]. A straight incision 3-to 5-cmlongwas made from the top of the greater trochanter toward the proximal side after satisfactory reduction. A rhombus-shaped awl was used to drill a hole at the front and middle 1/3 between the tip of the greater trochanter and the sinus piriformis. The medullary cavity was progressively expanded. The proximal femoral nail, which was matched with the femoral bone marrow cavity, was inserted. The nail end was placed parallel to the tip of the greater trochanter. The femoral neck screw and hip screw guide needle were inserted under X-ray fluoroscopy, and the guide needle was located approximately 5 mm below the femoral head. After ensuring accurate lateral and anteroposterior positioning, the proximal helical blades and a distal locking screw were inserted, and the incision was closed layer by layer. The PFNA material was provided by the Zhengda Company (Tianjin, China) and the Dabo Company (Xiamen, China).

### Postoperative treatment

For the patients in both groups, the drainage tube was removed within 12 h after operation. Second-generation cephalosporin antibiotics were administered to prevent infection, and alendronate (70 mg orally once/week), vitamin D3 for anti-osteoporosis therapy, and routinely applied analgesic drugs (celecoxib capsules, 120 mg orally twice per day) were given. Low molecular weight heparin (1500 μl subcutaneous injection once per day) was given to prevent deep vein thrombosis. The patients in the PFNA group began postoperative quadriceps muscle contraction and relaxation exercises on day 2, CPM training on day 3, active flexion and extension training of the hip and knee joint on day 7, walking with help without weight bearing on day 10, and gradually increased weight bearing according to fracture healing at 1–2 months. They could walk with weight bearing after confirmation of fracture healing at 4 months. The patients in the CPH group could walk with help at day 3 postoperatively. They underwent anteroposterior pelvic and lateral femoral X-ray examination at bedside on the first postoperative day to investigate the fracture’s reduction and the implant location.

The patients in the CPH group participated in hip flexion and extension exercises in bed on the first operative day, stood and walked with a crutch on the 2nd day after the operation, and gradually began walking independently after 2–3 weeks according to their condition. The patients in the PFNA group actively participated in quadriceps femuris muscle strengthening exercises as well as hip and knee flexion exercises within 2 weeks after the operation, performed non-weight-bearing activities with crutches after 2 weeks and gradually took on weight-bearing activities. After 1 month, 3 months or 6 months, depending on the condition of their fracture healing determined by X rays, they gradually began walking without crutches.

### Indicators and evaluating methods

The indexes, including operation time, blood loss (occult blood loss and total blood loss based on the Gross equation), weight training time, hospital stay, Harris score, American Association of Anesthesiologists grading standard (ASA) score, and complications such as deep vein thrombosis, pulmonary infections, urinary tract infections and bed sores, were observed (See Table 1 Patient Demographics).

**Table 1:** Demographics of patients with intertrochanteric fractures treated by PFNA and CPH in the elderly (n)

Group	Gender (M/F)	Age($\overline{x}$ ±s,year)	Course of disease($\overline{x}$ ±s,month)	ASA grade		Evans-Jensen classification		
				III	IV	III	IV	V
PFNA group	36/25	83.5 ± 4.8	27.9 ± 5.2	42	19	27	22	12
CPH group	27/20	83.8 ± 6.4	28.2 ± 6.9	30	17	17	19	11
χ2*/t* value		1.949	1.834	0.301	0.150	0.733	0.516	0.524
*P* value		0.054	0.069	0.583	0.699	0.693	0.473	0.469

Notes: PFNA stands for proximal femoral nail anti-rotation, CPH stands for cementless bipolar hemiarthroplasty, ASA stands for American Society of Anesthesiologists

### Statistical analysis

SPSS19.0 statistical software (International Business Machines Corporation, USA) was used for data analysis, and measurement data were expressed as the mean ± standard deviation. The unpaired *t* test was used for comparisons between the two groups, count data were analyzed using the χ^2^ test, and *P* < 0.05 was considered to indicate a significant difference.

## Results

**Fig. 1:**
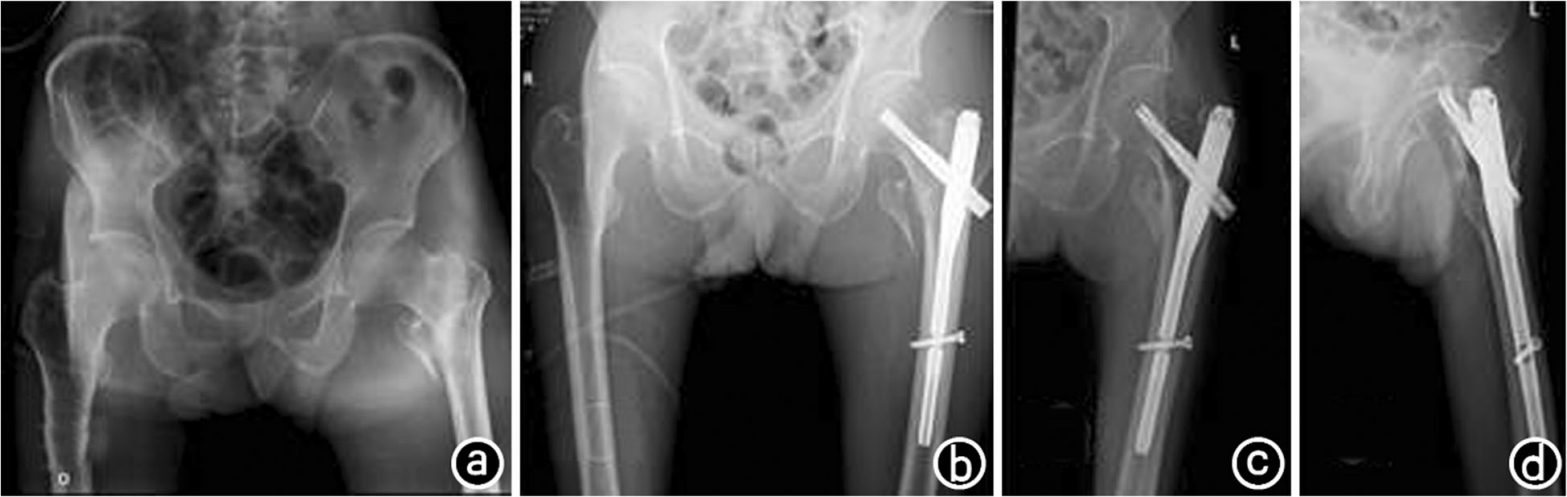
The patient was an 81-year-old male who accidentally fell on the ground while walking on March 25, 2012, causing left hip pain andlimited mobility. **a**. X-ray examination showed left comminuted intertrochanteric fracture and liberation of the great trochanter and lesser trochanter; **b**. Physical examination: adduction and internal rotation deformity was observed inhis left hip; left leg was approximately1.5 cm shorter than the right leg; percussion pain inhis large rotator and vertical percussion pain inhis limb. On March 27, 2012, he was treated with left intertrochanteric fracture fixation (PFNA) under nerve block anesthesia. Anteroposterior pelvis and lateral femoral examination after the operation showed good fracture alignment and satisfactory fixation; **c**, **d**. X-ray examination 2 years after the operation showed that the trochanteric fracture has healed without loosening or leakage of internal fixation

**Fig. 2:**
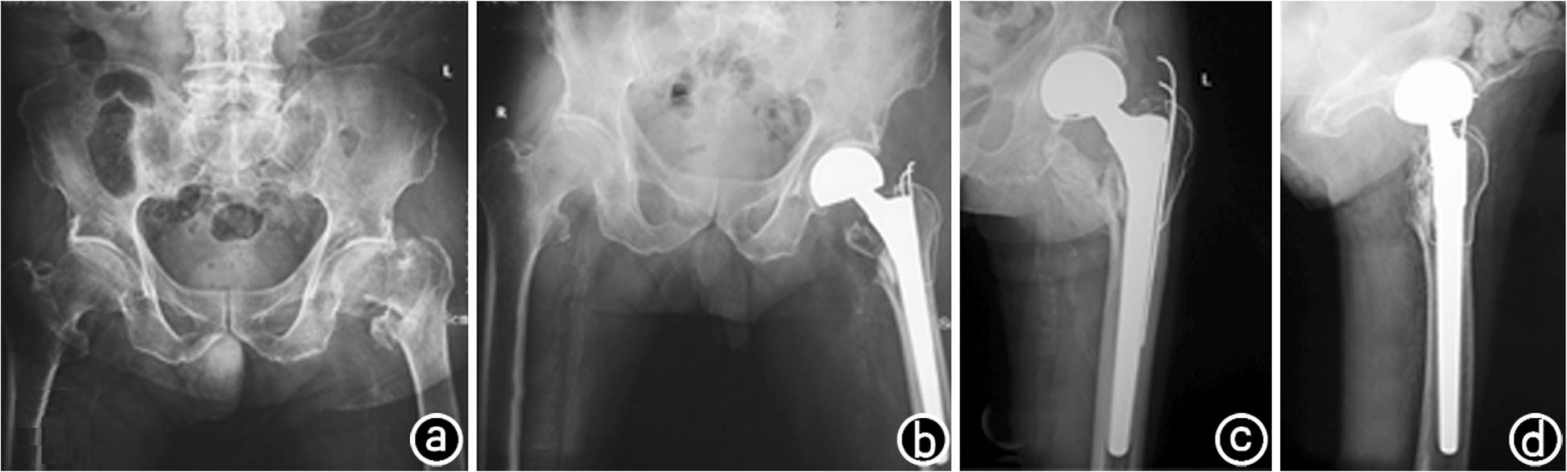
The patient was a 94-year-old male whofell from a height accidentally on November 12, 2011, causing left hip pain and limited activity. **a**. X-ray examination showed a left intertrochanteric comminuted fracture; **b**. Physical examination: mild adduction rotation deformity ofhis left hip; the left limb was approximately2cm shorter than the right limb. On November 15, 2011, he underwent left cementless bipolar hemiarthroplasty (CPH) under spinal anes thesia and Kirschner wire bunched fixation. Postoperative X-ray examination showed good positioning of the fracture and satisfactory positioning of the femoral head prosthesis and wire fixation; **c**, **d**. X-ray examination 2 years after the operation showed the left femoral head prosthesis in a good position, with no loosening or dislocation

All 108 patients were followed for 12–36 months, with an average of 12.5–36.2 months [(28.0 ± 6.3) months)]. The ASA scores of the two groups had no statistically significant difference. The mean operation time was (53.7 ± 15.2) min in the PFNA group and (77.5 ± 16.8) min in the CPH group, and the difference was significant (*P* < 0.05, Table 2). The average amount of blood loss was (132.5 ± 33.2) mL in the PFNA group and (286.3 ± 43.2) mL in the CPH group, and the difference was significant (*P* < 0.05, Table 2). The average postoperative weight training exercise time was (28.2 ± 3.7) days in the PFNA group and (3.1 ± 1.2) days in the CPH group, with significant differences between the two groups (*P* < 0.05, Table 2). The mean length of hospitalization stay was (7.6 ± 1.8) days in the PFNA group and (6.9 ± 2.2) days in the CPH group, and there was no statistically significant difference between the two groups (*P* > 0.05, Table 2). The Harris hip function score at 1 year after the operation was (87.7 ± 7.9) points in the PFNA group and (88.3 ± 9.2) points in the CPH group, with no statistically significant difference (*P* > 0.05, Table 3). Postoperative complications included urinary tract infection in three patients, pulmonary infection in two, deep vein thrombosis in three and bedsores in two in the PFNA group and complications include urinary tract infection in two patients, lung infection in three, deep vein thrombosis in four and bedsores in one in the CPH group (*P* > 0.05, Table 3). All the postoperative complications in both groups were actively treated and cured. Fixation loosening occurred in one patient in the PFNA group, and none occurred in the CPH group. Meanwhile, no patient in either group experienced hip varus or death. Typical cases are presented in Figs. 1, 2.

**Table 2:** Operation index and clinical efficacy of PFNA and CPH in treating intertrochanteric fractures in the elderly ($\overline{x}$ ±s)

Group	Cases (n)	Operation time (min)	Bleeding volume (ml)	Weight training time (d)	Hospital stay (d)	Harris score
PFNA group	61	53.7 ± 15.2	132.5 ± 33.2	28.2 ± 3.7	7.6 ± 1.8	87.7 ± 7.9
CPH group	47	77.5 ± 16.8	286.3 ± 43.2	3.1 ± 1.2	6.9 ± 2.2	88.3 ± 9.2
t value		7.71	20.93	46.70	1.82	0.36
*P* value		0.001	0.001	0.001	0.054	0.082

**Table 3:** Postoperative complications of PFNA and CPH in treating intertrochanteric fractures in elderly [n(%)]

Group	Urinary tract infection	Pulmonary infection	Deep vein thrombosis	Bedsore
PFNA group	3 (4.9%)	2 (3.3%)	3 (4.9%)	2 (3.3%)
CPH group	2 (4.3%)	3 (6.4%)	4 (8.5%)	1 (2.1%)
χ2 value	0.026	0.090	0.128	0.000
*P* value	> 0.05	> 0.05	> 0.05	> 0.05

## Discussion

Femoral intertrochanteric fracture refers to fractures between the base of the femoral neck and the lesser trochanter, and its incidence accounts for 3.57% of limb fractures [7]. It mainly occurs in the elderly, especially in patients over 75 years old. Elderly patients often have osteoporosis, poor fracture healing, complications resulting from being bedridden and high mortality [8]. Intertrochanteric fractures require surgical treatments, and objective and careful preoperative evaluations of the fracture are necessary for the development of a reasonable treatment plan [9]. Common intertrochanteric fracture treatments include intramedullary fixation (Gamma nail, PFNA), plate fixation (DHS, DCS) and CPH.

DHS and DCS have high bone condition requirements. Both of them are eccentric fixation with large torque and require great strength for screw fixation in biomechanics. The lateral plate of the DHS is located in the outer femur, and medial cortical defects of the femur may cause complications, including the screw cutting the femoral head, internal displacement or plate-side screw extrusion. Furthermore, DHS has other disadvantages, such as a long operation time and extensive bleeding, which means it is not ideal for elderly patients. Many elderly patients have osteoporosis, so the fixation effect is not often satisfactory [10, 11]. At present, most authors recommend PFNA and CPH as the first surgical choices for treating elderly patients with unstable intertrochanteric fractures [12, 13]. As a minimally invasive procedure, PFNA can maintain good biomechanical results and result inreliable fixation, making it a preferred technology for unstable intertrochanteric fractures associated with osteoporosis [14]. PFNA nails not only retain the advantages of Gamma nails, such as the short arm, reduced movement, and sliding compression but also increases the anti-rotation screw, which significantly enhances the anti-rotation, anti-compression and anti-tension abilities of the fracture end, increases the stability of the fracture end, and increased the uniformity of the bearing end force. Thus, it is particularly suitable for elderly patients with poor bone condition [15]. For elderly intertrochanteric fracture patients with Evans-Jensen type III or above, the femoral intertrochanteric fracture caused the loss important mechanical effects, such as support of the femoral neck, anti-rotation and anti- introversion. Intraoperative fracture reduction is difficult, and in femoral necks with serious osteoporosis, screw loosening and cutting are likely to occur. Studies have shown that the use of proximal femoral nails in the treatment of intertrochanteric fracture has a failure rate of 7.1–12.5% [16, 17]. In comparison, CPH can quickly restore hip function; it is mainly used to treat femoral neck fractures in the elderly, including unstable intertrochanteric fractures and failure of intertrochanteric fracture fixation [18]. Haentjens et al. [19] reviewed the relevant literature and noted that intertrochanteric comminuted fracture patients with severe osteoporosis may benefit from femoral head surgeries. CPH is recommended as a prior treatment for comminuted fractures with poor stability in the elderly severe osteoporosis, poor prognosis after internal fixation and a short life expectancy [20]. There is also controversial regarding the choice of cemented and cementless (biological) prostheses. For elderly patients with poor bone quality, a bone cement prosthesis can improve initial fixation strength, but a cementless prosthesis is conducive to biological fixation and can prevent cardiovascular toxicity caused by bone cement. Some studies have reported that with the improvement and development of implant design, materials and insertion techniques, the use of cementless prosthesis for artificial femoral replacement insenile patients with unstable intertrochanteric fractures can achieve better results compared with cemented prostheses [21]. Chuet al [10] used the Wagner stem prosthesis for hip replacement to treat unstable intertrochanteric fracture and obtained good results.

This study showed that the operation time and volume of intraoperative blood loss in the PFNA group were significantly lower than those of the CPH group (*P* < 0.05), and the postoperative ambulation exercise time in the CPH group was significantly shorter than that of the PFNA group (*P* < 0.05). The average hospital stay, Harris score at postoperative 1 year and postoperative complications had no significant differences between the two groups (*P* > 0.05). Postoperative complications included urinary tract infection in three patients, pulmonary infection in two, deep vein thrombosis in three and bedsores in two in the PFNA group and complications include urinary tract infection in two patients, lung infection in three, deep vein thrombosis in four and bedsores in one in the CPH group (*P* > 0.05, Table 3). All the postoperative complications in both groups were actively treated and cured. One case of fixation loosening occurred in the PFNA group, and none occurred in the CPH group. Meanwhile, no patient in either group experienced hip varus or death. PFNA offers theadvantages of micro-trauma, minimal bleeding and short operation times, while patients treated with CPH can begin functional exercise earlier. However, long-term follow-up results show that both procedures can reduce postoperative bedrest-related complications, obtain reliable fixation, relieve patients’ pain, and significantly improve patients’ quality of life.

We believe that both PFNA and CPH can obtain satisfactory clinical results in the treatment of intertrochanteric fractures in the elderly. However, clinicians should comprehensively assess the preoperative X-ray, CT and bone density test results, correctly classify the intertrochanteric fracture based on the Evens-Jensen type, and select reasonable surgical options. PFNA is suitable to treat unstable intertrochanteric fractures, but CPH is preferable for treating comminuted fractures in patients with severe osteoporosis, especially in the patients with trochanter fracture. We recommend the following indications for CPH for the treatment of intertrochanteric fracture: age > 75 years with severe osteoporosis; severe comminuted fracture; the presence of internal diseases and inability to tolerate long-term bed rest; implant failure or nonunion; femoral head disease; and voluntary arthroplasty. Its contraindications are as follows: severe medical illness, inability to tolerate surgery, potential sources of infection, and a life expectancy of less than two years. To ensure the length of the femoral prosthesis after implantation, it is necessary to apply a biological long-stem prosthesis the type of which should be selected based on the preoperative femoral index. Attempts should be made to reduce intertrochanteric fractures and fix them with wire bundling to obtain a more stable fixation. Elderly intertrochanteric fracture patients have reduced bone mass, andanti-osteoporosis medication should be provided during the perioperative period. Qiu et al. [22] conducted a randomized double-blind study of 77 patients with hip fracture with osteoporosis for 1 year and found that a lendronate had a satisfactory effect for treating osteoporosis. Therefore, surgical treatment combined with the administration of anti-osteoporosis drugs can increase patients’ bone density and bone quality and promote fracture healing to avoid cutting and fixation failure. Meanwhile, medical diseases should be actively treated during the perioperative period to optimize the patients’ state, increase the safety of surgery and reduce postoperative complications.

## Conclusion

In summary, CPH and PFNA are two safe and effective fixation methods for treating the elderly with intertrochanteric fractures for it can obtain stable fracture fixation, reduce pain, and restore function of the hip joint. However, hemiarthroplasty is less invasive and allows faster mobilization and recovery. Clinicians should strictly control surgical indications and choose the most effective internal fixation that is reasonable to obtain the most satisfactory clinical results. In the meantime, the study has limitations like small sample size of clinical cases and a retrospective study rather than prospective study, further study is needed in treatment regime.

## Data Availability

The data and materials are available from the medical records department of the General Hospital of Lanzhou Military Command. The datasets used and analysed during the current study are available from the corresponding author on reasonable request.

## Abbreviations

ASA: American Society of Anesthesiologists
CPH: Cementless bipolar hemiarthroplasty
IMN: Intramedullary nailing
PFNA: Proximal femoral nail anti-rotation
